# Verified Research Productivity Among Matched Orthopaedic Surgery Residency Applicants: Establishing a National Baseline and Comprehensive Analysis

**DOI:** 10.7759/cureus.98930

**Published:** 2025-12-10

**Authors:** Adrian Bozocea, Bryce R Michael, Amy Y Zhao, Diego Garcia, Nicholas Siegel, Amil R Agarwal, Savyasachi C Thakkar, Dawn LaPorte, Gregory Golladay

**Affiliations:** 1 Department of Orthopaedic Surgery, Augusta University Medical College of Georgia, Augusta, USA; 2 Department of Orthopaedic Surgery, University of Arizona College of Medicine - Tucson, Tucson, USA; 3 Department of Orthopaedic Surgery, The George Washington University School of Medicine, Washington DC, USA; 4 Department of Orthopaedic Surgery, Johns Hopkins Health System, Baltimore, USA; 5 Department of Orthopaedics, Virginia Commonwealth University School of Medicine, Richmond, USA

**Keywords:** bibliometric analysis, clinical research productivity, electronic residency application services (eras), medical education, orthopaedic surgery, publication threshold, publication verification, pubmed database, residency application, surgical education

## Abstract

Introduction: The shift of the United States Medical Licensing Examination (USMLE) Step 1 to pass/fail scoring in 2022 elevated research productivity's role in orthopaedic surgery residency applications. With the 2027 Electronic Residency Application Service (ERAS) cycle reforms limiting research entries to peer-reviewed publications and consolidating repeated presentations, establishing verifiable research productivity baselines is critical. This study provides the earliest comprehensive verification of PubMed-indexed publications across an entire national orthopaedic match cohort, quantifying disparities between self-reported and verified activities and establishing benchmarks to assess future reform impacts.

Methods: Cross-sectional bibliometric analysis examined first-year orthopaedic surgery residents who matched in the 2023-2024 National Resident Matching Program (NRMP) or military cycle (N=929, 97% of matched positions). A novel automated disambiguation system integrated institutional affiliation matching, name uniqueness, and temporal filtering to verify publications prior to September 24, 2023, yielding 4,774 verified publications from 46,897 extracted. Programs were assigned to six tiers based on composite scores integrating established program rankings with contemporary research productivity. Tier 1 (10 programs, score ≥ 50) and Tier 6 (76 programs, score < 2) represented the highest and lowest tiers. Statistical analyses employed Kruskal-Wallis and Mann-Whitney U tests for group comparisons, receiver operating characteristic (ROC) analysis for threshold determination, and incremental benefit analysis to assess diminishing returns.

Results: Of 958 identified positions, 929 residents were collected (773 with complete data). The mean verified publication count was 5.5 ± 9.0 (median 3.0; range 1-139), representing 23% of mean self-reported outputs (23.8) in the 2024 NRMP Charting Outcomes report; 77% comprised non-peer-reviewed activities. Clinical research constituted 46% (n=2187) of publications, with original research accounting for 75% (n=3566). Residents were first, second, or third author on 68.2% (n=3254) of publications. Higher-tier programs had significantly greater publication counts (Tier 1: 9.7 ± 8.8 vs. Tier 6: 3.7 ± 6.7; H=100.54, p < 0.0001). ROC analysis identified an optimal threshold of two to four publications (AUC = 0.66) for predicting high-tier matches. Incremental benefit analysis revealed the largest marginal gain between one and two publications (mean tier improvement=0.55; p < 0.001), with diminishing returns thereafter, and a secondary benefit peak at seven publications (0.80; p < 0.05).

Conclusions: This study establishes the earliest verified baseline for research productivity in an orthopaedic surgery match cohort, documenting substantial inflation between self-reported activities and peer-reviewed publications. Positioned between the 2022 Step 1 pass/fail transition and the 2027 ERAS application reforms, these data provide benchmark metrics for evaluating the impact of future verification systems and application changes. Identification of optimal thresholds (two to four papers) and quantification of incremental benefits offer evidence-based guidance for students, advisors, and program directors. Our novel verification methodology, achieving 83.2% (n=773 residents) cohort coverage, establishes a reproducible framework applicable across specialties for transparent scholarly productivity assessment.

## Introduction

The transition of the United States Medical Licensing Examination (USMLE) Step 1 examination to a pass/fail scoring system on January 26, 2022, fundamentally altered the competitive landscape of orthopaedic surgery residency applications. This policy change eliminated a longstanding quantitative metric that residency programs historically utilized for initial applicant screening and ranking decisions. With the loss of Step 1 as a numerical differentiator, program directors have increasingly emphasized alternative measures of academic achievement and scholarly potential, particularly research productivity as quantified through publications, presentations, and abstracts.

The magnitude of this shift is evident through the National Resident Matching Program (NRMP) Charting Outcomes. In 2022, successfully matched orthopaedic surgery residents reported authoring a mean of 16.5 abstracts, presentations, and publications [1]. By 2024, merely two years following the Step 1 scoring modification, this figure escalated to 23.8 research outputs, representing a 44.2% increase [2]. This highlights both an emphasis on the importance of research as an orthopaedic surgery resident applicant, as well as a possible misrepresentation or inflation of research being reported, not only raising questions about the nature, quality, and verifiability of reported scholarly activities, but also raising broader concerns about equity, accessibility, and the potential for an unsustainable "research arms race" among medical students pursuing competitive surgical specialties.

In response to evolving concerns about application transparency and the meaningful assessment of scholarly contributions, the Association of American Medical Colleges (AAMC) announced comprehensive reforms to the 2027 ERAS (Electronic Residency Application Service) application cycle [3]. Beginning with applications opening in June 2026, the MyERAS platform will revise the Research and Scholarly Work section to limit it to only publications and presentations that have undergone the peer-review process, condense posters and presentations presented in multiple venues under the same thematic project to a single entry, distinguish three projects as most meaningful, and link Scholarly Work to Experiences. These reforms explicitly aim to better capture the quality and impact of applicants' academic contributions beyond the simple enumeration of activities.

The temporal positioning of the current study - capturing the 2024 match cohort situated between the 2022 Step 1 pass/fail implementation and the forthcoming 2027 ERAS reforms - provides a unique opportunity to establish baseline metrics of verified research productivity. These data will serve as essential reference points for assessing the impact of future verification systems, evaluating trends in scholarly inflation, and measuring whether enhanced application scrutiny produces meaningful changes in reporting accuracy. Furthermore, this baseline enables longitudinal tracking of how orthopaedic surgery residency competitiveness evolves in response to policy interventions across the medical education continuum.

To address these questions and establish rigorous benchmarks, we conducted a comprehensive national bibliometric analysis of PubMed-indexed publications authored by first-year orthopaedic surgery residents from the 2024 match cycle. We developed and implemented a novel automated verification system capable of disambiguating author identities across institutional affiliations, achieving 83% coverage of all matched positions. Our analysis characterized publication type, authorship position, research domain, institutional affiliation, and specialty-specific relevance while assessing associations between verified research productivity and residency program competitiveness. In this comprehensive analysis, we investigated the publishing rate, publication characteristics, impact of residency program tier, optimal publication thresholds, and incremental publication benefits.

We hypothesized that despite the reported 44.2% increase in total research outputs following Step 1 pass/fail implementation, the number of verifiable, peer-reviewed publications indexed in PubMed would demonstrate substantially lower growth, suggesting that the observed increase predominantly reflects non-peer-reviewed scholarly activities. This study aims to provide objective, verified data to inform medical students pursuing orthopaedic surgery, faculty advisors counseling applicants, and program directors evaluating scholarly productivity within the context of holistic residency selection.

## Materials and methods

This study was deemed exempt by our institutional review board.

Residents

We collected data on orthopaedic surgery residents from the 2024 match class using publicly accessible NRMP program directories, Department of Defense medical education training site directories, orthopaedic department websites, and social media platforms [4-6]. All publicly available orthopaedic surgery interns were collected, including military and international students. From 218 programs participating in both the NRMP and ERAS, we identified 916 positions offered with 915 positions filled; however, only 213 programs with 891 total residents displayed publicly available information, representing 97% of non-military programs as of 2024. For programs participating in the military match, 12 programs with 43 total residents were identified; however, only nine programs with 38 total residents displayed publicly available information, representing 88% of military programs as of 2024.

**Figure 1:**
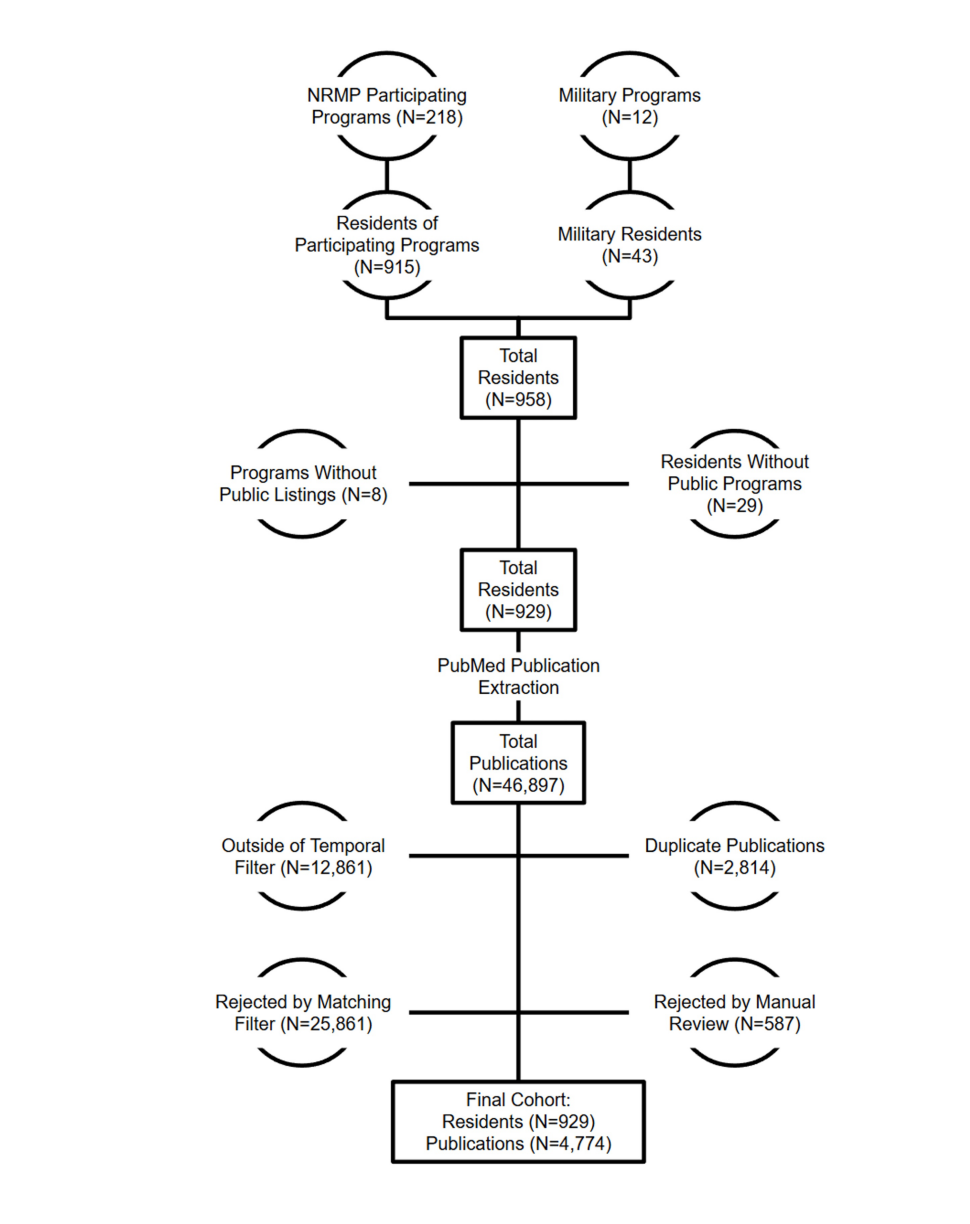
Schematic showing sample selection from the 2024 orthopaedic residency match NRMP, National Resident Matching Program

Of the 958 positions identified, eight programs (29 positions) did not provide public listings of their 2024 resident class. Among residents with available information, 86 lacked public medical school affiliations, and 396 lacked undergraduate institution data (Figure 1).

Publications

We systematically extracted publication data from PubMed, the largest online database of biomedical literature, for each resident in our cohort. To ensure accurate attribution, we first standardized all resident names, program names, medical school names, and undergraduate institution names. We then established terms for programs, medical schools, undergraduate institutions, types of papers, types of research, and medical specialties to facilitate comprehensive searching and categorization.

All PubMed searches were restricted to publications before September 24, 2023, to ensure only research completed prior to residency application submission was included. Publications were matched to residents based on standardized author names and institutional affiliations. We excluded any publications without overlapping institutional affiliations to minimize false attributions.

For each author, we performed a comprehensive PubMed search, retrieving all associated author publications with a maximum limit of 1,000 publications per author. Publication metadata was concurrently collected, including PMID, title, MeSH terms, author position, publication date, journal, and affiliations. Based on metadata, each publication underwent multi-dimensional classification using a hierarchical keyword-matching algorithm to identify three independent taxonomies: (1) publication type (e.g., case report, meta-analysis, original research), (2) medical research type (e.g., clinical, basic science, translational), and (3) medical specialty (encompassing 38 medical specialties).

Data quality and completeness

The location-based dataset exhibited substantial missingness across three variables: additional degrees (97.4%), undergraduate institution (41.8%), and medical school (8.0%). Additional degrees measured additional self-reported professional degrees (e.g., PhD, JD, PharmD, DMD, MPH, BSN).

Complete data were observed for all geographic variables (program, city, state, and tier), participant identifiers (name and year of residency), and publication metrics. The high completeness rate for critical variables ensured robust statistical analyses, while the persistent missingness in additional degrees was deemed acceptable, as this variable was not central to our primary analyses.

Residency program rankings

To assess the relationship between resident research productivity and program prestige, we utilized the ranking system introduced by Jones et al., which ranks programs based on total citations from 2005 to 2015 in 45 orthopaedic journals [4]. We supplemented this with our own composite ranking system, incorporating both Jones rank data and contemporary citation metrics. Our ranking methodology employed min-max normalization to transform total citations (range: 0-40,122; mean: 4,323; median: 1,983) and total publications (range: 0-2,579; mean: 283; median: 143) to a 0-100 scale. A composite score was calculated using 60:40 weights for citations and publications, respectively.

Programs were stratified into six tiers based on composite score thresholds derived from natural breakpoints in the score distribution - Tier 1: Composite Score ≥ 50, Tier 2: Composite Score ≥ 20 and < 50, Tier 3: Composite Score ≥ 10 and < 20, Tier 4: Composite Score ≥ 5 and < 10, Tier 5: Composite Score ≥ 2 and < 5, and Tier 6: Composite Score < 2.

Publication matching and disambiguation

After data collection, to ensure appropriate statistical analyses, an automated publication-filtering system was developed in Python (version 3.12.3; Python Language Reference, Python Software Foundation, Wilmington, DE) using the pandas, NumPy, and SciPy libraries to match and verify publications for each resident and their respective program [8]. The system employed a multi-component scoring algorithm that integrated a name uniqueness analysis, institutional affiliation matching, domain-specific relevance assessment, and temporal filtering. Name uniqueness was evaluated with integration across publicly available Social Security Administration birth name records, US Census surname frequencies, and the names-dataset library for international coverage, calculating Fellegi-Sunter probabilistic weights, inverse document frequency (IDF) scoring, and entropy-based metrics, which were combined into a composite score normalized via a sigmoid transformation. Cultural pattern detection using regular expression matching and surname lookup tables adjusted component weights based on naming conventions (e.g., Hispanic compound surnames, East Asian surname patterns), improving accuracy across diverse populations. Institutional affiliation matching employed the FuzzyWuzzy library for Levenshtein distance-based string comparison between author affiliations and known program keywords, with tiered scoring thresholds (>90: 0.9, >80: 0.8, >70: 0.7). Domain relevance was assessed through the detection of 120+ orthopaedic-specific terms in titles, matching against 60+ specialty journals, and evaluation of MeSH terminology, with each component contributing up to 0.25 points.

These components were combined into a confidence score using weighted aggregation (name uniqueness: 0.20, affiliation: 0.45, domain relevance: 0.35), with acceptance thresholds dynamically adjusted based on name commonality, ranging from 0.2 for highly unique names to 0.6 for very common names. Override rules automatically accepted publications with perfect affiliation matches combined with any orthopaedic indicator, or high domain relevance (≥0.35) with strong affiliation scores (≥0.7). Publications near decision boundaries (within 0.1 of the threshold), those with conflicting signals between domain relevance and affiliation matching, and accepted publications for very common names (uniqueness < 0.3) were flagged for manual review. System accuracy was assessed through manual verification of all flagged publications, with discrepancies resolved by author consensus.

Statistical analyses

All statistical analyses were performed using Python version 3.12.3 with the scipy.stats, numpy, statsmodels, matplotlib, seaborn, and pandas libraries [8]. Statistical significance was set at α = 0.05 for all tests. Statistical significance was indicated using standard conventions (*p < 0.05, **p < 0.01, ***p < 0.001). Publications were included for statistical analyses if they met the following criteria: (1) published between January 2011 and September 2024, (2) contained a valid PubMed ID (PMID), and (3) could be reliably attributed to a resident in our database. Duplicate publications were identified and removed based on PMID.

Four primary categorical variables were analyzed: Program Tier, Author Position (numerical position within author lists), Research Type (clinical, basic science, epidemiological, diagnostic, pharmacological, other), and Paper Type (original research, methodology, review, case report, letter, systematic review, meta-analysis), with Number of Publications serving as the connecting continuous variable across all categorical determinations. For each variable, we computed frequency distributions, cumulative percentages, and summary statistics with coverage percentages exceeding 95%.

To address potential name-matching failures resulting in false zeros, we implemented a conservative imputation strategy. For programs with any residents with zero publications and where >50% of residents had publications (n=80), we imputed the 10th percentile of non-zero publication counts and assigned these to residents with zero publications, matching pre-imputation publication distributions. For programs with zeros and where <50% of residents had publications (Total = 12 programs, Tier 2 = 1 program, Tier 4 = 1 program, Tier 5 = 3 programs, Tier 6 = 7 programs), tier-specific minimum values were applied (Tier 2: 2 publications, Tiers 3-6: 1 publication). This approach balanced statistical considerations with the recognition that some programs may have low publication expectations. This conditional approach increased mean publications by 2-7% across tiers, preserving the competitive gradient while avoiding over-correction. Results were validated against univariate statistical and tier-based minimum approaches, showing strong convergence.

Descriptive Statistics

Continuous variables were summarized using means (SD) and medians (IQR). Categorical variables were presented as frequencies and percentages. The distribution of publications was visualized using histograms and examined for normality using visual inspection.

Group Comparisons

For comparisons of publication counts and tier distributions across publication groups, the non-parametric Kruskal-Wallis H test was employed due to the non-normal nature of publication rate data and the ordinal nature of tier rankings. Post-hoc pairwise comparisons were conducted using Mann-Whitney U tests (for Kruskal-Wallis), and a Bonferroni correction was applied to correct for multiple data comparisons between tiers.

Threshold Analysis

To identify publication thresholds where tier differences became non-significant, we systematically tested thresholds from 1 to 20 publications. For each threshold, residents were dichotomized into groups (< threshold vs. ≥ threshold), and tier distributions were compared using Mann-Whitney U tests. Effect sizes were calculated as r = Z/√N. The threshold analysis was supplemented by examining sample sizes at each threshold to ensure adequate statistical power.

Receiver Operating Characteristic (ROC) Analysis

ROC curves were constructed to evaluate the discriminative ability of publication counts for predicting high-tier residency matches (defined as tier ≤ median tier). Statistical significance of the area under the curve (AUC) was assessed using the DeLong method, which provides a nonparametric variance estimation for the AUC, with a two-tailed z-test comparing the observed AUC against random chance (0.5). Bootstrap validation (2,000 iterations) was performed to corroborate the confidence interval. The optimal threshold was determined using Youden's J statistic (J = sensitivity + specificity - 1). A "near-optimal" range was defined as thresholds achieving ≥ 95% of the maximum J value, acknowledging the practical equivalence of thresholds with similar discriminative ability.

Incremental Benefit Analysis

To assess diminishing returns, we calculated the incremental benefit of each additional publication as the difference in mean tier between residents with n publications versus n-1 publications. The statistical significance of incremental benefits was tested using independent-samples t-tests.

Sensitivity Analyses

All primary analyses were conducted on both the original dataset (including zeros) and the imputed dataset. Additionally, analyses excluding outliers (>75 publications) were performed to ensure robustness of findings. Results were consistent across these approaches unless otherwise noted.

Ethics statement

This study did not involve human participants, identifiable personal data, or animals. The analysis was based exclusively on publicly available data, and Institutional Review Board approval and informed consent were not required.

Code availability

The Python code for the automated publication-filtering system and statistical analyses is available from the corresponding author upon reasonable request.

## Results

Research productivity of the 2024 orthopaedic residency match cohort

**Figure 2:**
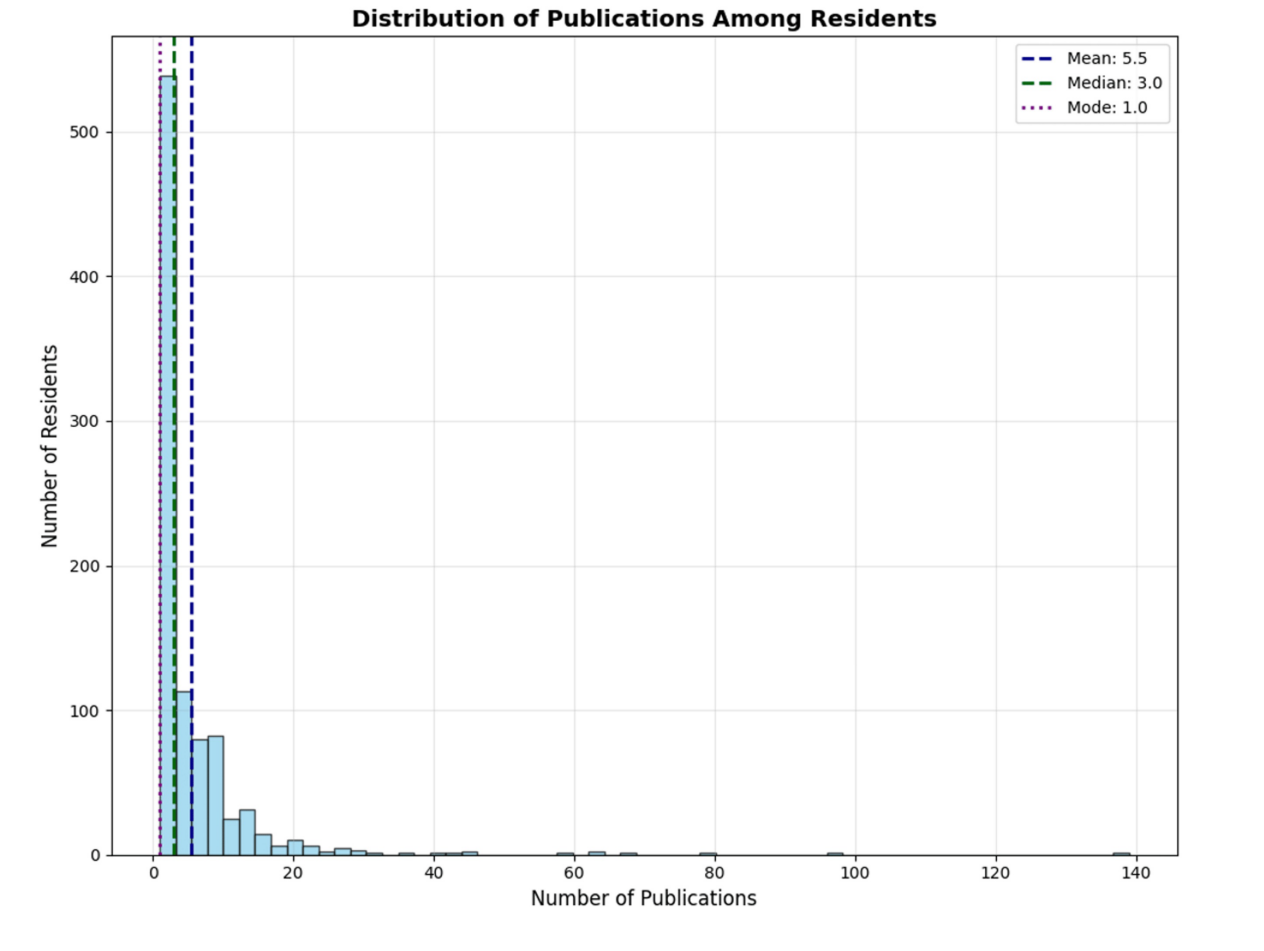
Number of 2024 orthopaedic residents by number of publications

Our cohort of 929 residents authored a mean of 5.5 ± 9.01 peer-reviewed publications (median: 3.0, range: 1-139) prior to residency application (Figure 2). The distribution of publications was positively skewed, with 260 residents (28.0%) having one publication, 283 (30.5%) having two to three publications, 247 (26.6%) having four to nine publications, and 139 (15.0%) having 10 or more publications (Table 1). Regarding authorship position, residents were first authors on 1365 publications (28.6%), second authors on 1077 (22.6%), and third authors on 812 (17.0%) (Table 1).

**Table 1:** Categorization of authorship from the 2024 orthopaedic match (total residents = 929, total publications = 4774)

Authorship Variable	N (%)
Residents with 1 Publication	260 (28.0%)
Residents with 4-9 Publications	247 (26.6%)
Residents with ≥10 Publications	139 (15.0%)
1st Author Publications	1365 (28.6%)
2nd Author Publications	1077 (22.6%)
3rd Author Publications	812 (17.0%)
4th Author Publications	561 (11.8%)

Characteristics of research publications

Our cohort collectively authored 4774 peer-reviewed publications before September 24, 2023. Of these, 3566 (74.7%) were original research publications, 672 (14.1%) were reviews and meta-analyses, and 332 (7.0%) were case reports (Table 2). The majority of publications were clinical research (2187, 45.8%), followed by other research types (1567, 32.8%), public health/epidemiological studies (588, 12.3%), and basic science research (292, 6.1%) (Table 2). Orthopaedic-specific publications comprised 1754 (36.7%) of the total, with an additional 2148 (45.0%) in non-surgical fields and 872 (18.3%) in surgical fields The top five subject areas accounted for 79.6% of all publications: orthopaedic surgery (36.3%), geriatrics (15.2%), general surgery (10.5%), internal medicine (9.2%), and pediatrics (5.8%) (Table 2).

**Table 2:** Categorization of publications from the 2024 orthopaedic match (total publications = 4774)

Publication Categorization/Variable	N (%)
Original Research	3566 (74.7%)
Review/Systematic Review/Meta-Analysis	672 (14.1%)
Case Report	332 (7.0%)
Clinical	2187 (45.8%)
Other	1567 (32.8%)
Public Health/Epidemiological	588 (12.3%)
Basic Science	292 (6.1%)
Orthopaedic	1754 (36.7%)
Surgical	872 (18.3%)
Nonsurgical	2148 (45.0%)
Total	100.0% (4774)

Research productivity by program tier

Based on our composite ranking system, 41 (4.4%) residents matched into tier-1 programs, 168 (18.1%) into tier-2 programs, 142 (15.3%) into tier-3 programs, 146 (15.7%) into tier-4 programs, 139 (15.0%) into tier-5 programs, and 293 (31.5%) into tier-6 programs.

**Figure 3:**
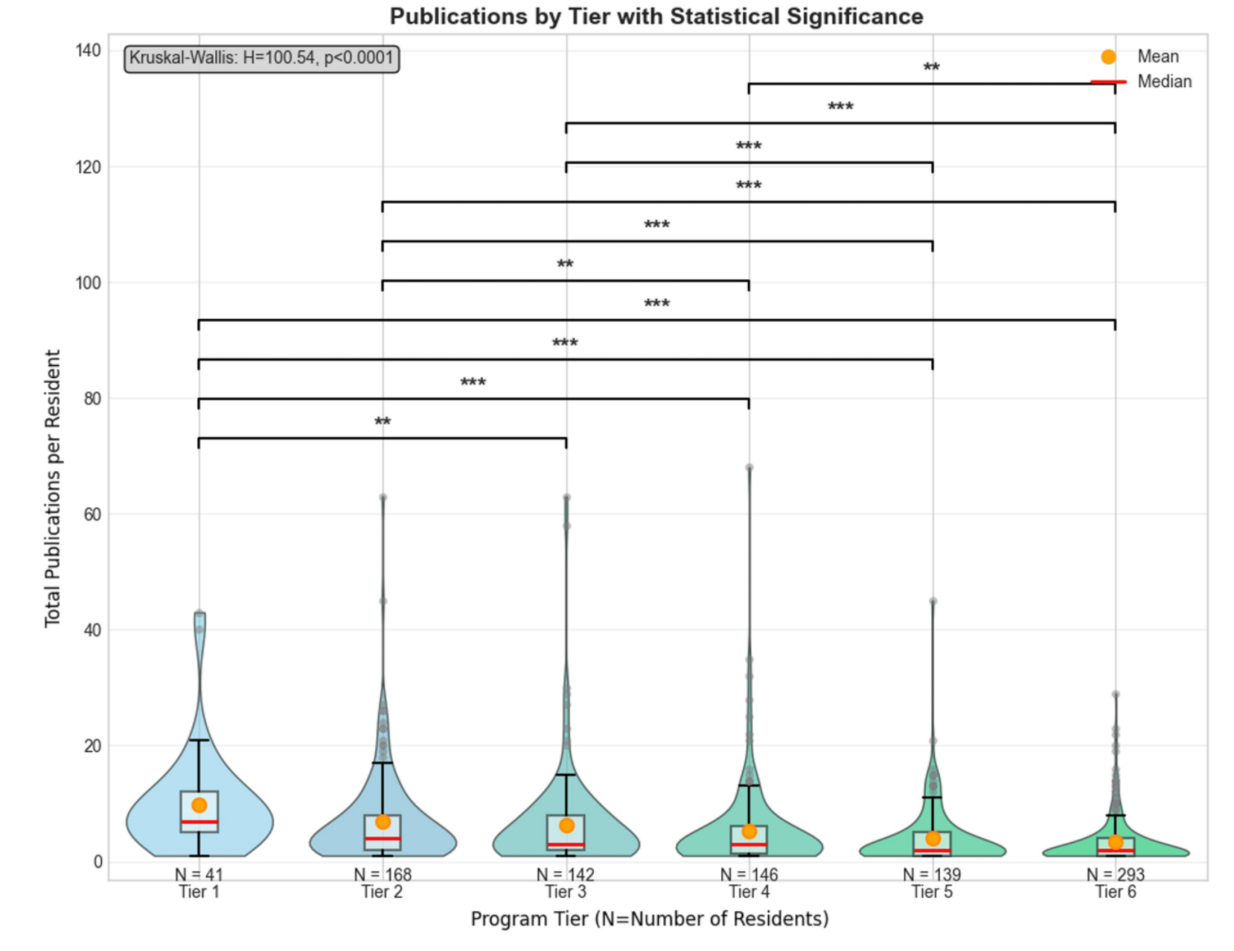
Violin plot showing publications between program tiers Publication counts > 75 were excluded for visualization purposes. Violin plots display the distribution with box plot elements: lower/upper whiskers (minimum/maximum), box edges (Q1/Q3), red line (median), orange marker (mean), and gray circles (outliers). N indicates the number of residents within each orthopaedic program tier. Programs were tiered 1-6, with Tier 1 as the most competitive, based on a composite score threshold measuring program research productivity. The Kruskal-Wallis test showed significant differences across tiers (H = 100.54, p < 0.0001). Post-hoc pairwise comparisons using the Mann-Whitney U test revealed significant differences between program tiers (**p < 0.01 and ***p < 0.001).

Programs ranked highly by the composite system were assigned lower numerical values (i.e., the first-ranked program was given the numerical value of 1). Research productivity differed significantly across program tiers (Kruskal-Wallis: H = 100.54, p < 0.0001). Research productivity data are depicted using violin plots because the data are non-parametric and positively skewed (Figure 3).

Tier-1 programs had residents with the highest publication counts (mean: 9.7 ± 8.8, median: 7), followed by a progressive decrease with tier-6 programs earning the lowest publication counts (mean: 3.7 ± 6.7, median: 2). Post-hoc pairwise comparisons using Mann-Whitney U revealed several significant differences between tiers. Tier 1 programs showed significantly higher publication counts compared to both Tier 5 (U = 4,444, p < 0.001) and Tier 6 programs (U = 9,720, p < 0.001). Tier 2 (U = 34,529, p < 0.001) and Tier 3 (U = 27,818, p < 0.001) programs had significantly more publications than Tier 6 programs. Additional significant differences were observed between Tiers 1-4 (p < 0.01), Tiers 2-5 (p < 0.01), Tiers 3-5 (p < 0.01), and Tiers 4-6 (p < 0.05), all following the pattern of higher-ranked programs demonstrating greater scholarly productivity among their residents.

Program tier by research productivity of residents

**Figure 4:**
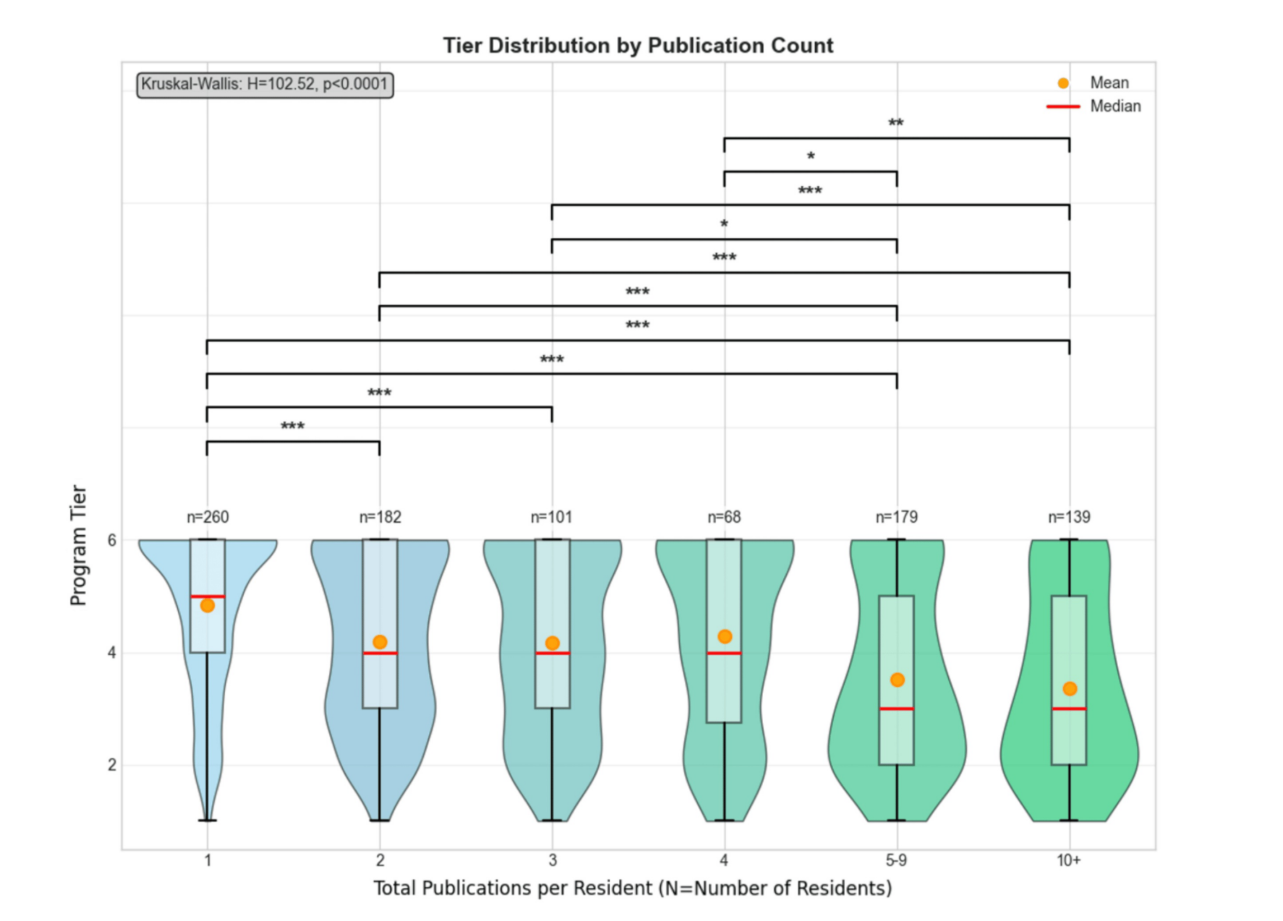
Violin plot showing program tiers between publication rates Violin plots display the distribution with box plot elements: lower/upper whiskers (minimum/maximum), box edges (Q1/Q3), red line (median), and orange marker (mean). N indicates the number of residents within each publication tier. Programs were tiered 1-6, with Tier 1 as the most competitive, based on a composite score threshold measuring program research productivity. The Kruskal-Wallis test showed significant differences across total publications (H = 102.52, p < 0.0001). Post-hoc pairwise comparisons using the Mann-Whitney U test revealed significant differences between publication tiers (*p < 0.05, **p < 0.01, and ***p < 0.001).

We analyzed the distribution of program tiers based on resident publication counts (Figure 4). The Kruskal-Wallis test revealed significant differences in tier distribution across publication count groups (H = 102.52, p < 0.0001). As illustrated in Figure 4, there was a clear pattern showing that residents with higher publication counts were more likely to be from higher-ranked (lower-numbered) tier programs. The mean program tier increased progressively with increasing publication productivity. Residents with only one publication had a mean tier of 4.84 ± 1.40, while those with two, three, and four publications had mean tiers of 4.20 ± 1.50, 4.17 ± 1.57, and 4.28 ± 1.66, respectively. Notably, residents with five to nine publications showed a substantial increase to a mean tier of 3.53 ± 1.63, and those with 10 or more publications had the highest mean tier of 3.37 ± 1.63.

Post-hoc pairwise comparisons using Mann-Whitney U tests revealed multiple significant differences. Residents with only one publication were from significantly lower-ranked programs compared to those with two publications (U = 29,519, p < 0.001), three publications (U = 16,280, p < 0.001), five to nine publications (U = 33,524, p < 0.001), and 10 or more publications (U = 26,924, p < 0.001). Similarly, residents with two publications were from significantly lower-ranked programs than those with five to nine publications (U = 20,142, p < 0.001) and 10 or more publications (U = 16,329, p < 0.001). Residents with three (U = 8,943, p < 0.001) and four (U = 6,147, p < 0.01) publications were also from significantly lower-ranked programs than those with 10 or more publications. No significant differences were observed between residents with five to nine vs. 10 or more publications. This finding complements our previous analysis and reinforces the strong association between program competitiveness and research productivity. Residents who publish prolifically (≥10 publications) are disproportionately concentrated in Tiers 1-3, while those with minimal publication output (one to two publications) are more evenly distributed across all tiers, with higher representation in Tiers 4-6.

Threshold analysis for publication impact

**Figure 5:**
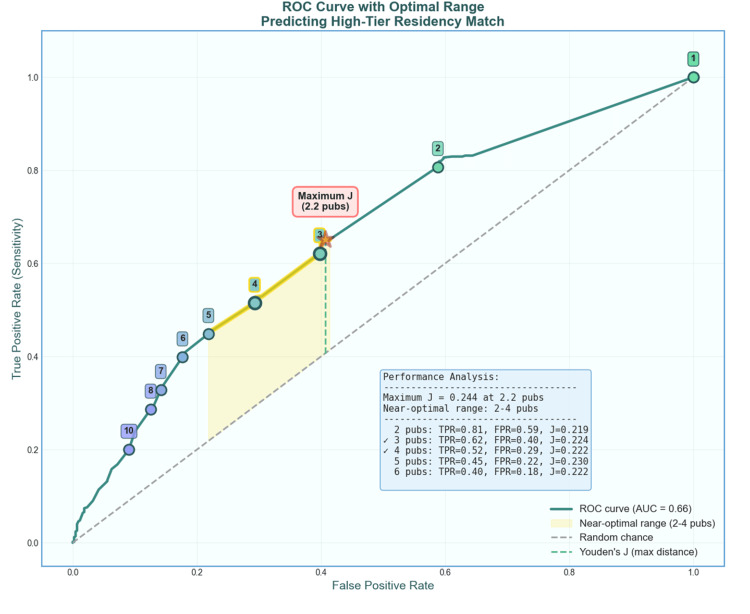
Receiver operating characteristic (ROC) curve for predicting high-tier residency match based on publication count The curve (AUC = 0.66, 95% CI: 0.63–0.70, p < 0.001 vs. chance) shows the trade-off between sensitivity and false-positive rate at different publication thresholds (numbered circles, 0-10 publications). The yellow-highlighted region indicates the near-optimal threshold range (two to four publications) where Youden's J statistic is ≥ 95% of its maximum value. The red star marks the point of maximum Youden's J (0.244) at 2.2 publications. The dashed green line illustrates Youden's J as the maximum vertical distance from the diagonal reference line (random chance). Among integer thresholds, 5 achieved the highest Youden’s J (0.23). The inset table shows performance metrics for thresholds within and adjacent to the optimal range.

To determine the optimal publication threshold for predicting match success in high-tier programs (Tiers 1-3), we performed ROC curve analysis (Figure 5). The AUC was 0.66 (95% CI: 0.63-0.70), indicating performance significantly greater than random chance in distinguishing applicants who matched into high-tier programs (DeLong test: z = 9.11, p < 0.001; bootstrap validation with 2,000 iterations corroborated the confidence interval). The curve exhibited a characteristic concave shape with improved sensitivity at lower publication thresholds, suggesting that even modest research productivity provides discriminatory value.

Using Youden's J statistic to identify the optimal balance between sensitivity and specificity, we found the maximum J value of 0.244 occurred at 2.2 publications. To account for the practical constraints of integer publication counts and the statistical uncertainty inherent in threshold selection, we identified a near-optimal range defined as thresholds achieving ≥95% of the maximum J value. This analysis revealed a threshold range of two to four publications as near-optimal for predicting high-tier residency matches.

Within this range, the performance metrics demonstrated favorable sensitivity with acceptable false-positive rates. At the two-publication threshold, the model achieved a true positive rate (TPR) of 0.81 with a false positive rate (FPR) of 0.59 (J = 0.219). The three-publication threshold yielded TPR = 0.62 and FPR = 0.40 (J = 0.224), while four publications resulted in TPR = 0.52 and FPR = 0.29 (J = 0.222). Notably, when examining integer thresholds, five publications provided the highest Youden's J value (0.230).

**Figure 6:**
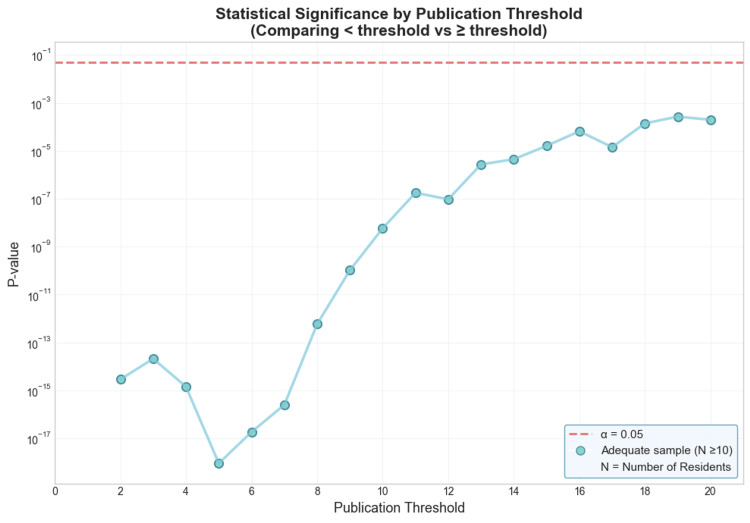
Statistical significance of tier differences by publication threshold P-values (log scale) from Mann-Whitney U tests comparing academic tiers between residents with fewer than the threshold publication versus those above the threshold, for thresholds ranging from 1 to 20. The horizontal dashed line indicates α = 0.05. N indicates the number of residents within a threshold. Filled circles represent thresholds with adequate sample sizes (N ≥ 10) in the higher publication group. For thresholds 1-20, all thresholds maintained adequate sample sizes. The connected line shows the trend across thresholds where valid comparisons were possible.

To determine the publication count at which tier differences between residents become non-significant, we conducted Mann-Whitney U tests comparing academic tiers between residents with fewer than a publication threshold versus those above the publication threshold, for thresholds ranging from 1 to 20 publications (Figure 6). Across all tested thresholds (1-20), we found statistically significant differences in academic tier between the two groups (all p < 0.05). This pattern indicates that even at the highest publication threshold examined, residents with 20 or more publications occupied significantly different program tiers compared to those with fewer publications.

Incremental benefit analysis

**Figure 7:**
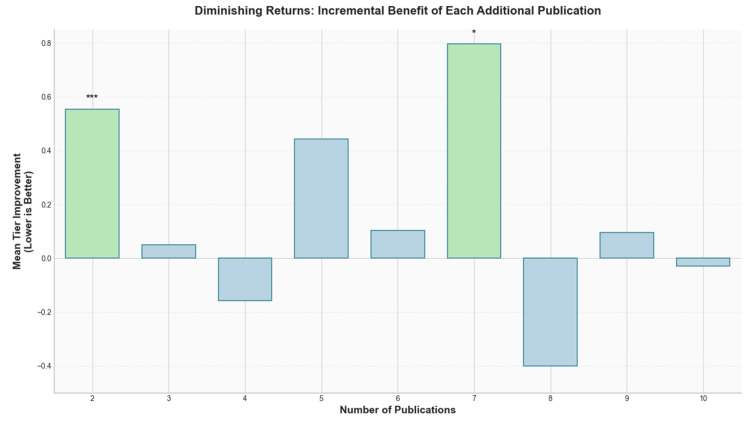
Incremental benefit of each additional publication Bar graph displaying the mean tier improvement (lower values indicate better tier placement) associated with each additional publication from 2 to 10. The y-axis represents mean tier improvement, with negative values indicating worse tier placement; bars show the incremental benefit, and the green-highlighted bars at two and seven publications are marked with an asterisk. * and *** indicate statistical significance at p < 0.05 and p < 0.001, respectively, based on independent-samples t-tests comparing residents with N publications versus N-1 publications.

To evaluate the marginal utility of each additional publication, we conducted an incremental benefit analysis examining the mean tier improvement associated with increasing publication counts from 1 to 10 publications (Figure 7). The analysis revealed a clear pattern of diminishing returns, with the most substantial benefits occurring early in the publication trajectory.

The transition from one to two publications yielded the most significant incremental benefit, with a mean tier improvement of 0.55 (n = 182, p < 0.001), representing over half a tier advancement on average. Following this substantial initial gain, the incremental benefits decreased markedly, with the third publication providing only a 0.05 tier improvement (n = 101, p > 0.05) and the fourth publication showing a slight negative effect (-0.16, n = 68, p > 0.05).

An unexpected secondary peak emerged at the seventh publication threshold, where we observed the largest increase in incremental benefit (0.80 tier improvement, n = 27, p < 0.05). However, this benefit was not sustained, as the eighth and 10th publications showed negative incremental effects (-0.40 and -0.03, respectively) with a small positive effect at the ninth publication (0.10, n = 24, p > 0.05).

## Discussion

Our cross-sectional bibliometric analysis of 929 first-year orthopaedic surgery residents revealed that the mean number of PubMed-indexed publications (5.5 ± 9.0, median 3.0) was markedly lower than the self-reported research outputs documented in the 2024 NRMP's Charting Outcomes reports (23.8). Residents who matched into higher-tier programs, as defined by Jones et al.'s [4] citation-based composite rankings, demonstrated significantly higher publication counts than their lower-tier counterparts (all p < 0.001). ROC curve analysis identified a near-optimal threshold range of 2-4 publications for distinguishing high-tier matches (AUC = 0.66, maximum Youden's J = 0.244 at 2.2 publications). The most significant incremental benefit occurred between one and two publications (mean tier improvement = 0.55), with diminishing returns thereafter and a secondary benefit peak observed at seven publications (0.80). These findings suggest modest gains early in the publication count, with diminishing returns thereafter. These results reinforce and extend prior bibliometric work by Toci et al., who similarly noted that a single publication conferred an advantage but that additional publications yielded limited further benefit [9].

The pronounced disparity between self-reported research outputs and verified PubMed publications suggests that approximately 77% of "research" listed on applications comprises non-peer-reviewed activities, such as abstracts, presentations, and institution-level projects. This inflation mirrors concerns in other specialties. For example, neurosurgery applicants reported an average of 13.5 research experiences, compared with a bibliometrically validated range of 4.8-6.6 publications [10-13], while otolaryngology matched residents demonstrated a similar gap between reported and indexed outputs [14]. These systematic patterns suggest that current application reporting mechanisms inadequately distinguish between peer-reviewed scholarship and other research-related activities, potentially disadvantaging applicants with verified high-impact publications whose total "research activity" counts appear numerically lower than peers reporting predominantly non-indexed work.

Our analysis of publication type and research category revealed that clinical research accounted for the plurality (46%) of indexed publications, with original research accounting for 75%, reviews/meta-analyses for 14%, and case reports for 7%. Despite only 37% of publications being orthopaedic-specific, we found no significant difference in match tier based on specialty focus, suggesting that programs value the overall rigor of scholarship over a narrow subspecialty focus. This aligns with previous observations in surgical fields that program directors prioritize methodological quality and journal impact over topic specificity [15]. The substantial representation of publications in geriatrics, general surgery, internal medicine, and pediatrics - collectively accounting for 40.7% of output beyond orthopaedic surgery - indicates that successful applicants pursue diverse scholarly interests during medical school while developing domain expertise applicable across multiple clinical contexts.

Temporal significance and establishment of baseline metrics

The unique temporal positioning of this study - capturing the 2024 match cohort between the January 26, 2022, Step 1 pass/fail transition and the forthcoming 2027 ERAS application reforms - provides baseline data for assessing the longitudinal impact of policy interventions on residency application dynamics. The 44.2% increase in reported research outputs between 2022 (16.5) and 2024 (23.8) occurred entirely within the post-Step 1 pass/fail era, suggesting that elimination of numerical scoring intensified emphasis on alternative quantitative metrics [1,2]. Our verification demonstrates that this reported increase substantially reflects the proliferation of non-indexed activities rather than peer-reviewed publications, raising concerns about whether heightened research emphasis produces meaningful scholarly development or primarily incentivizes the enumeration of low-barrier activities.

The 2027 ERAS reforms explicitly aim to improve transparency and meaningful assessment of academic contributions. Our baseline verification data will enable future researchers to determine whether these structural changes reduce reporting inflation, improve concordance between reported and verified outputs, or alter the competitive landscape of orthopaedic surgery applications. Furthermore, as additional specialties transition, our methodological framework provides a reproducible template for establishing specialty-specific baselines and tracking longitudinal trends.

Methodological innovation and reproducibility across specialties

A significant contribution of this study extends beyond orthopaedic surgery-specific findings to establish a methodological framework for rigorous verification applicable across graduate medical education. Our automated disambiguation system, integrating name uniqueness analysis, institutional affiliation matching, and temporal filtering, achieved coverage of an entire national match cohort, exceeding typical bibliometric study coverage rates. The system's incorporation of cultural pattern detection for diverse naming conventions and dynamic threshold adjustment based on name commonality addresses longstanding challenges in large-scale author disambiguation.

This methodology offers several advantages for medical education research. First, the reproducible framework enables longitudinal tracking within specialties and cross-specialty comparisons of verified research productivity. Second, the high coverage rate minimizes the selection bias inherent in studies that rely on smaller samples or specific institutional contexts. Third, the automated approach with manual review flagging provides scalability for future annual cohort analyses while maintaining accuracy. As residency selection increasingly emphasizes research productivity across specialties, standardized verification methodologies become essential for establishing evidence-based benchmarks, detecting temporal trends, and assessing the impact of policy interventions on application dynamics. Our system's code and disambiguation algorithms can be adapted for other specialties seeking to establish comparable baseline data.

Study strengths

Strengths of this study include its comprehensive national scope, standardized disambiguation of author identities through institutional affiliation matching, and the application of advanced statistical methods (ROC analysis, incremental threshold testing) to define actionable publication targets. By leveraging PubMed, the most widely used biomedical database, we ensure broad coverage and applicability of our findings. The tiered program ranking using Jones et al.'s citation metrics adds another layer of validity by correlating resident productivity with departmental scholarly impact [4].

Study limitations

However, this study includes several limitations. First, despite rigorous name-affiliation matching, some publications may have been missed due to name changes or incomplete affiliation data, potentially underestimating true productivity. Some publications may also have been missed due to publications in journals not indexed by PubMed. Second, 156 residents (16.8%) lacked complete publication data, leading to possible selection bias if their productivity differed systematically; additionally, eight programs (29 residents) lacked public listings. Although this only represented 3.1% of the final cohort, these individuals could have exacerbated bias if they differed systematically. Third, the AUC of 0.66 for publication count indicates modest discriminatory power, suggesting that research output alone is insufficient as a sole predictor of program tier. Fourth, other unmeasured applicant factors, such as clinical performance, letters of recommendation, Step 2 CK scores, and interviews, undoubtedly contribute to match outcomes and could confound the association between publications and program tier. Finally, this cross-sectional design captures a single match cycle, precluding assessment of longitudinal trends or causal inferences about the impact of Step 1 pass/fail implementation on individual applicant behavior.

Future research should establish longitudinal cohorts tracking verified publication rates across multiple match cycles to assess whether the 2027 ERAS reforms produce measurable changes in reporting accuracy and application dynamics. Incorporating measures of publication quality, such as journal impact factors, citation metrics, and h-indices, may further refine competitive benchmarks beyond simple enumeration. Qualitative investigations evaluating program director perspectives on research quality versus quantity, including the relative weight assigned to different publication types, could inform evidence-based guidance for applicants and advisors. Extension of this verification methodology to additional competitive specialties would enable cross-specialty comparisons and the identification of specialty-specific research expectation patterns. Finally, analysis of the relationship between verified research productivity and subsequent residency performance metrics, including board examination scores, research productivity during training, and career outcomes, would provide crucial data regarding the predictive validity of pre-residency scholarship for surgical training success.

## Conclusions

This study establishes the earliest comprehensive verified baseline of research productivity for an entire national orthopaedic surgery match cohort, documenting substantial disparities between self-reported scholarly activities and verifiable peer-reviewed publications. Our findings reveal that approximately 77% of reported research outputs comprise non-peer-reviewed activities, highlighting critical challenges in application transparency and equitable assessment of scholarly merit. Uniquely positioned between the 2022 Step 1 pass/fail transition and the forthcoming 2027 ERAS application reforms, these baseline data provide essential benchmarks for evaluating the longitudinal impact of policy interventions on residency selection dynamics. The substantial inflation between reported activities and verified publications documented here empirically validates the rationale underlying the 2027 ERAS reforms, which address information asymmetries through peer-review restrictions, presentation consolidation, and mechanisms for highlighting meaningful scholarly contributions. These structural changes may reduce reporting inflation and improve concordance between reported and verified outputs, effects that can now be assessed using the verification methodology and baseline thresholds established in this study.

Beyond immediate implications for application reform, this work provides evidence-based guidance for medical students and advisors by identifying optimal publication thresholds (two to four peer-reviewed papers) and characterizing incremental benefits of research productivity, potentially enabling more strategic allocation of scholarly effort during medical training. From a broader medical education perspective, the modest discriminatory power of publication counts (AUC = 0.66) confirms that research output alone provides insufficient predictive value for match outcomes, supporting holistic review approaches that incorporate clinical performance, scores, letters, and extracurriculars alongside scholarly productivity. This finding reinforces that research requirements should complement, rather than supersede, the assessment of attributes directly relevant to medical training and practice. The reproducible verification methodology established here offers a template applicable across medical specialties for establishing standardized benchmarks, tracking longitudinal trends, and informing evidence-based policy decisions regarding research expectations in graduate medical education.
